# Restoring mitochondrial DNA copy number preserves mitochondrial function and delays vascular aging in mice

**DOI:** 10.1111/acel.12773

**Published:** 2018-05-09

**Authors:** Kirsty Foote, Johannes Reinhold, Emma P. K. Yu, Nichola L. Figg, Alison Finigan, Michael P. Murphy, Martin R. Bennett

**Affiliations:** ^1^ Division of Cardiovascular Medicine University of Cambridge Cambridge UK; ^2^ MRC Mitochondrial Biology Unit University of Cambridge Cambridge UK

**Keywords:** aging, mitochondria, mitochondrial DNA, vascular stiffness

## Abstract

Aging is the largest risk factor for cardiovascular disease, yet the molecular mechanisms underlying vascular aging remain unclear. Mitochondrial DNA (mtDNA) damage is linked to aging, but whether mtDNA damage or mitochondrial dysfunction is present and directly promotes vascular aging is unknown. Furthermore, mechanistic studies in mice are severely hampered by long study times and lack of sensitive, repeatable and reproducible parameters of arterial aging at standardized early time points. We examined the time course of multiple invasive and noninvasive arterial physiological parameters and structural changes of arterial aging in mice, how aging affects vessel mitochondrial function, and the effects of gain or loss of mitochondrial function on vascular aging. Vascular aging was first detected by 44 weeks (wk) of age, with reduced carotid compliance and distensibility, increased β‐stiffness index and increased aortic pulse wave velocity (PWV). Aortic collagen content and elastin breaks also increased at 44 wk. Arterial mtDNA copy number (mtCN) and the mtCN‐regulatory proteins TFAM, PGC1α and Twinkle were reduced by 44 wk, associated with reduced mitochondrial respiration. Overexpression of the mitochondrial helicase Twinkle (Tw^+^) increased mtCN and improved mitochondrial respiration in arteries, and delayed physiological and structural aging in all parameters studied. Conversely, mice with defective mitochondrial polymerase‐gamma (PolG) and reduced mtDNA integrity demonstrated accelerated vascular aging. Our study identifies multiple early and reproducible parameters for assessing vascular aging in mice. Arterial mitochondrial respiration reduces markedly with age, and reduced mtDNA integrity and mitochondrial function directly promote vascular aging.

## 1 INTRODUCTION

Aging of the large conduit arteries is a major cause of morbidity and mortality, contributing to hypertension (high blood pressure) and stroke. Arterial aging is associated with multiple structural and functional changes, including vessel dilatation and wall thickening, loss of vascular smooth muscle cells (VSMCs) and elastin, deposition of collagen, endothelial dysfunction and low‐grade inflammation. In turn, these changes (which are present in both humans (Wang et al., 2007) and rodents (Wang et al., 2006)) result in vessel stiffening. Vascular stiffening may also be a consequence of hypertension, in part because of stress‐induced matrix synthesis by mechanosensitive cells, resulting in a positive feedback loop (reviewed in Humphrey, Harrison, Figueroa, Lacolley & Laurent, 2016).

Large artery aging has been demonstrated extensively in humans, and several invasive and noninvasive parameters of vascular stiffness can reliably predict cardiovascular events (reviewed in Vlachopoulos, Aznaouridis & Stefanadis, 2010). However, dissecting the molecular mechanisms underlying vascular aging requires studies in animal models, and similar studies in rodents are more difficult to perform. Currently, it is unclear what the earliest time points that constitute vascular aging in laboratory mice are, which physiological measures of large artery stiffness correspond most closely to humans, and whether similar processes underlie changes in mechanical properties in mouse and human arteries. Aging research is time‐consuming and expensive because of the long time courses needed. Therefore, identifying the earliest time points that show the most sensitive and reproducible changes and parameters is crucial in obtaining scientific consensus for mouse models of vascular aging.

Mitochondria contain multiple copies of mitochondrial DNA (mtDNA) that encode ribosomal and transfer RNAs and many essential proteins required for oxidative phosphorylation. Loss of mtDNA integrity by both altered mitochondrial DNA copy number (mtCN) and increased mutations is implicated in cellular dysfunction with aging (Szklarczyk, Nooteboom & Osiewacz, 2014). Reactive oxygen species (ROS), many of which are generated by mitochondria, also increase with age. However, the role of mitochondria in aging may extend beyond ROS, and it is unclear whether decreased mitochondrial function promotes vascular aging directly or is just a consequence of aging.

We examined multiple parameters of vascular function, histological markers, and markers of mitochondrial damage and function during normal vascular aging, and the effects of reducing or augmenting mitochondrial function on the onset and progression of vascular aging. We identify early, standardized time points and reproducible physiological parameters for vascular aging studies in mice. Vascular aging begins at far earlier time points than previously described in mice, with compliance, distensibility, stiffness and pulse wave velocity (PWV) being the best discriminators for normal aging and manipulations. mtCN and mitochondrial respiratory function are reduced when functional and structural manifestations of vascular aging begin. Rescue of the mtCN deficit observed in normal aging improves mitochondrial respiration and delays all parameters of vascular aging, while reduced mtDNA integrity accelerates vascular aging. Together these data highlight the direct role of mtDNA‐mediated mitochondrial dysfunction in the progression of vascular aging.

## 2 RESULTS

### 2.1 Physiological markers of normal aging in mice

**Figure 1:**
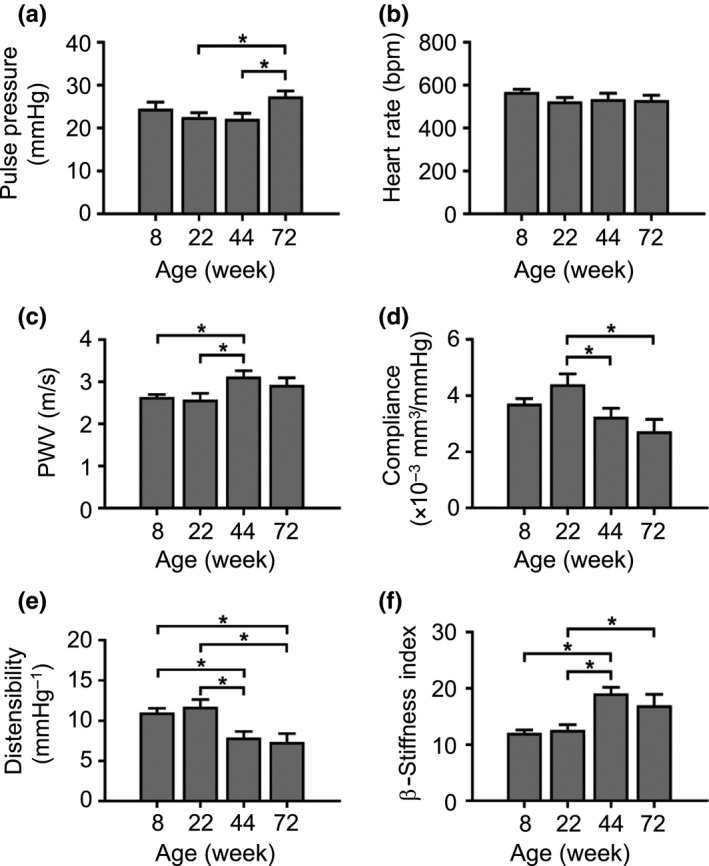
Functional evidence that mice develop vascular stiffness by 44 wk of age. (a–f) Pulse pressure, heart rate, aortic pulse wave velocity (PWV), carotid artery compliance, carotid artery distensibility and carotid artery β‐stiffness index in WT mice aged 8–72 wk. Data are means ± *SEM*. *p < .05 using ANOVA with Tukey post‐test (*n* = 10–17)

The hallmark of vascular aging is vascular stiffening, predominantly due to loss of elastin and elevated collagen deposition. Vessel stiffening is challenging to measure in mice, and there is a lack of consensus about the most sensitive and discriminatory parameters. We analysed multiple indices of stiffness of the common carotid artery and aorta during normal mouse aging, including compliance (change in volume (ΔV)/change in pressure (ΔP)), distensibility (change in arterial diameter (ΔD) or circumferential area in systole and diastole) and β‐stiffness index (ΔP per ΔD). ΔV, ΔD and Doppler flow velocities were assessed by imaging the common carotid artery (Figure S1) and ΔP by intra‐arterial blood pressure measurement. PWV is considered the gold standard measure of vascular stiffness in humans (Vlachopoulos et al., 2010) and was measured in mice by simultaneous measurement of aortic pressure at two different anatomical sites (Figure S2). To determine the earliest time points that demonstrate changes in vascular physiological parameters, we examined wild‐type (WT) C57BL/6J mice at 8, 22, 44 and 72 wk of age. We did not observe any differences in any parameter between male and female mice, and therefore, both sexes were used. Mean systolic and diastolic blood pressure did not change between 8 and 72 wk (Figure S3a,b). Pulse pressure was similar from 8 to 44 wk, but increased significantly by 24% at 72 wk (Figures 1a and S3c), while heart rate did not change at any age (Figure 1b). In contrast, aortic PWV was significantly elevated at 44 wk (Figure 1c). Similarly, carotid arterial compliance and distensibility were significantly reduced by 44 wk, with a concomitant increase in β‐stiffness index at the same age (Figure 1d–f). Systolic and diastolic Doppler flow velocities were also elevated at 72 wk by 36% and 37%, respectively (Figure S3d,e).

### 2.2 Changes in vascular structure in normal vascular aging

**Figure 2:**
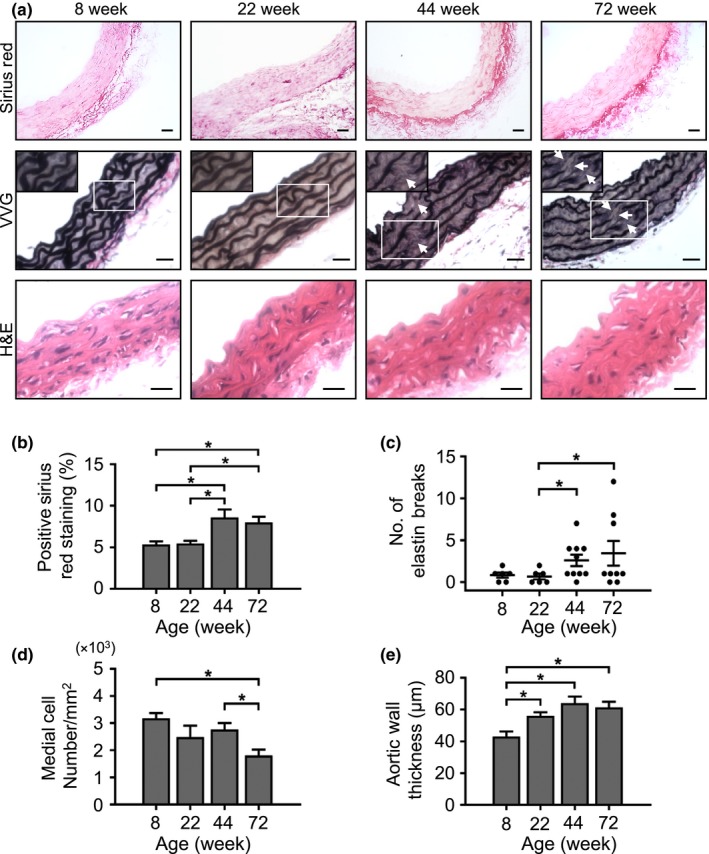
Structural evidence that mice develop increased collagen, elastin breaks, reduced cellularity and vessel thickening by 44 wk of age. (a) Representative images of aorta sections stained with Sirius Red, Verhoeff–van Gieson (VVG) or haematoxylin and eosin (H&E). Examples of elastin breaks are indicated by the white arrows. Scale bars = 25 μm. (b) Collagen quantification using Sirius Red‐positive staining, (c) number of elastin breaks quantified by VVG staining, (d) medial cellularity and (e) aortic wall thickness. Data are means ± *SEM*. *p < .05 using ANOVA with Tukey post‐test (*n* = 4–9 mice)

Vascular aging in humans is associated with reduced VSMC cellularity, elastin breaks, increased collagen deposition, and intimal, medial and adventitial thickening. However, it is unclear whether these changes occur in mice, and whether they precede, accompany or follow the functional effects of aging. Medial collagen content increased significantly by 1.6‐fold at 44 wk and remained elevated at 72 wk, with a characteristic periadventitial accumulation (Figure 2a–b). Elastin breaks increased 4.3‐fold over the same time frame, which increased a further 1.2‐fold by 72 wk (Figure 2a,c). Medial cell number was unchanged between 8 and 72 wk, but medial cellularity decreased 1.4‐fold between 44 and 72 wk, while overall aortic thickness increased 1.3‐fold at 22 wk, but did not change thereafter (Figure 2a,d,e). Inflammation markers examined by immunohistochemistry showed that the percentage of IL‐1β‐positive cells was unchanged from 8 to 44 wk, but increased 1.5‐fold at 72 wk. There was no consistent trend in VCAM‐1 expression (Figure S4), and macrophages were infrequent (<1% cells).

### 2.3 Effects of aging on mtCN and mitochondrial respiration

**Figure 3:**
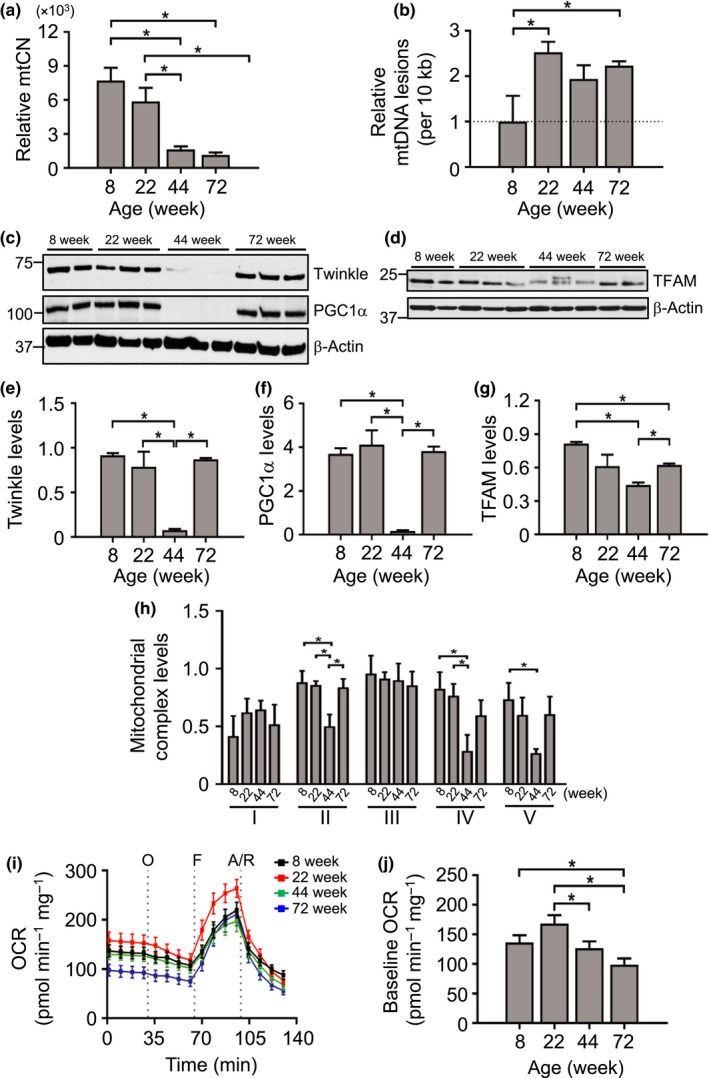
Reductions in vascular mitochondrial DNA copy number, mitochondrial protein expression and mitochondrial respiration with age in mice. (a) Relative aortic mitochondrial copy number (mtCN) (*n* = 4–6 mice), (b) relative aortic mtDNA lesions, (c–g) Western blots showing protein levels of the major mtCN regulators Twinkle, PGC1α or TFAM with their respective quantifications (relative to β‐actin), (h) relative protein expression levels of mitochondrial complexes (I–V relative to citrate synthase levels) quantified from Western blot, (i) Seahorse oxygen consumption rate (OCR) in intact aortas indicating injection points (grey dotted lines) of oligomycin (O), FCCP (F), antimycin A and rotenone (A/R) and (j) quantified baseline OCR (*n* = 3 aortas per group). Data are means ± *SEM*. *p < .05 using ANOVA with Tukey post‐test

Cells contain many copies of the mitochondrial genome, and although mtCN declines with age in some cells (Mengel‐From et al., 2014), the rate of decline and the relationship between mtCN and mitochondrial respiration vary markedly between tissues (Wachsmuth, Hubner, Li, Madea & Stoneking, 2016). We examined mtCN relative to nuclear DNA in aortas from mice aged 8–72 wk. Relative aortic mtCN was unchanged between 8 and 22 wk, but decreased significantly at 44 and 72 wk (Figure 3a). mtDNA integrity was reduced at 22 wk and remained reduced thereafter, indicating relatively increased mtDNA lesions (Figures 3b and S5a). This mtDNA damage was not due to the presence of the mouse equivalent of the human 4977‐bp “common deletion,” as this was unchanged across all ages (Figure S5b).

mtDNA synthesis is directly regulated by a number of proteins, including the mitochondrial transcription factor A (TFAM), mitochondrial helicase Twinkle and peroxisome proliferator‐activated receptor‐gamma coactivator 1‐alpha (PGC1α), which is an upstream mediator of TFAM expression and mitochondrial mass. Reductions in mtCN at 44 wk were mirrored by marked reductions in Twinkle, TFAM and PGC1α protein expression (Figure 3c–g), and similar reductions in Twinkle and TFAM mRNA at 44 wk (Figure S5c,d). mtDNA encodes many of the mitochondrial electron transport chain complex proteins, and there was a significant decline in complexes II, IV and V at 44 wk with recovery of complex II at 72 wk (Figure 3h). To determine how changes in mtCN and complex expression affect mitochondrial function, we measured mitochondrial respiration in intact aorta tissue using a Seahorse XFe24 Bioanalyzer at baseline and after inhibition of specific complexes with oligomycin A (complex V) or rotenone/antimycin A (complex I/III), or uncoupling with carbonyl cyanide‐*4*‐(trifluoromethoxy)phenylhydrazone (FCCP). Baseline oxygen consumption rate (OCR) was unchanged between 8 and 22 wk, but was reduced at 44 and 72 wk (Figure 3i,j). In contrast, extracellular acidification rate (ECAR), a measure of glycolysis, was unchanged between 8 and 72 wk (Figure S5e,f). These data show that despite a possible compensatory rise in the expression of PGC1α, TFAM and Twinkle (and a rise in nuclear‐encoded complex II) at 72 wk, this was not sufficient to rescue mtCN and mitochondrial respiration at 72 wk.

### 2.4 Effects of augmented mitochondrial respiration on vascular aging markers

Our data show that physiological and structural markers of vascular aging can be detected early in mice, and are most marked at 44 wk of age, with some additional changes between 44 and 72 wk. mtCN and mitochondrial respiration decline over the same ages, with reduced expression of mtCN‐regulatory proteins and mitochondrial complexes at 44 wk. However, it is unknown whether the decline in mtCN and reduced mitochondrial function are a cause or a consequence of vascular aging, or whether mtCN itself has direct effects on mitochondrial function. We therefore examined markers of vascular aging in gain‐ and loss‐of‐function mice that have increased mtCN or mtDNA damage, respectively.

We used mice overexpressing the mitochondrial helicase Twinkle to determine whether augmenting mtCN and mitochondrial respiration delays vascular aging. The nuclear‐encoded mitochondrial helicase Twinkle, the mitochondrial polymerase‐gamma (PolG) and mitochondrial single‐strand DNA binding proteins (mtSSB) form the mtDNA replisome machinery. Twinkle unwinds short stretches of dsDNA in the 5′–3′ direction in preparation for mtDNA replication, and alterations in Twinkle expression lead to changes in mtCN (Jemt et al., 2011). Twinkle^‐/‐^ mice show multiple mtDNA deletions and develop progressive respiratory dysfunction and chronic late‐onset mitochondrial disease (Tyynismaa et al., 2005). In contrast, mice overexpressing Twinkle show increased mtCN (Tyynismaa et al., 2004), which could potentially increase mitochondrial respiration. Indeed, Twinkle overexpression can rescue single‐nucleotide variants and replication stalling of mtDNA, reduce ROS‐induced apoptosis and improve tissue function (Pohjoismaki et al., 2013), with no change in ROS (Yu et al., 2017). We used mice heterozygous for overexpression of the WT Twinkle transgene (Tw^WT+^; denoted hereafter as Tw^+^), as very high mtCN in homozygous Twinkle mice can result in respiratory chain deficiency (Ylikallio, Tyynismaa, Tsutsui, Ide & Suomalainen, 2010).

**Figure 4:**
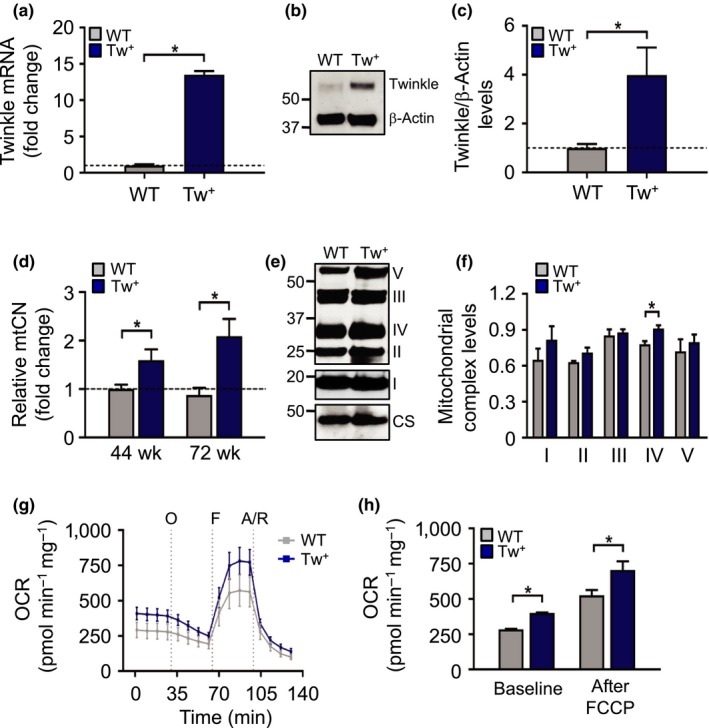
Tw^+^ mice have increased vascular mtCN and enhanced mitochondrial respiration. (a) qPCR for *Twinkle* mRNA (*n* = 3), (b–c) Western blot for Twinkle protein in aortas of WT and Tw^+^ mice at 44 wk (*n* = 3–4), (d) mitochondrial copy number (mtCN) in aorta in WT and Tw^+^ mice at 44 and 72 wk (*n* = 5–6), (e–f) representative Western blot of mitochondrial complexes (I–V) in aortas from 72‐wk‐old WT and Tw^+^ mice with quantified levels relative to citrate synthase (CS) (*n* = 3–5), (g) Seahorse profile of WT and Tw^+^ mouse aortas at 44 wk indicating injection of the compounds (grey dotted lines) oligomycin (O), FCCP (F) and antimycin A and rotenone (A/R), with (h) Quantification of OCR at baseline and after addition of the uncoupler FCCP (*n* = 2 aortas). WT (grey labels); Tw^+^ (blue labels). Data are means ± *SEM*. *p < .05 using ANOVA with Tukey post‐test

Tw^+^ mice had increased *Twinkle* mRNA and Twinkle protein expression in aortas compared to WT mice (Figure 4a–c), with increased mtCN evident up to late time points (Figure 4d). Although only complex IV protein levels were significantly increased in Tw^+^ mice (Figure 4e,f), Tw^+^ mice showed a marked increase in aortic mitochondrial respiration at baseline and after FCCP at 44 wk (Figure 4g,h).

**Figure 5:**
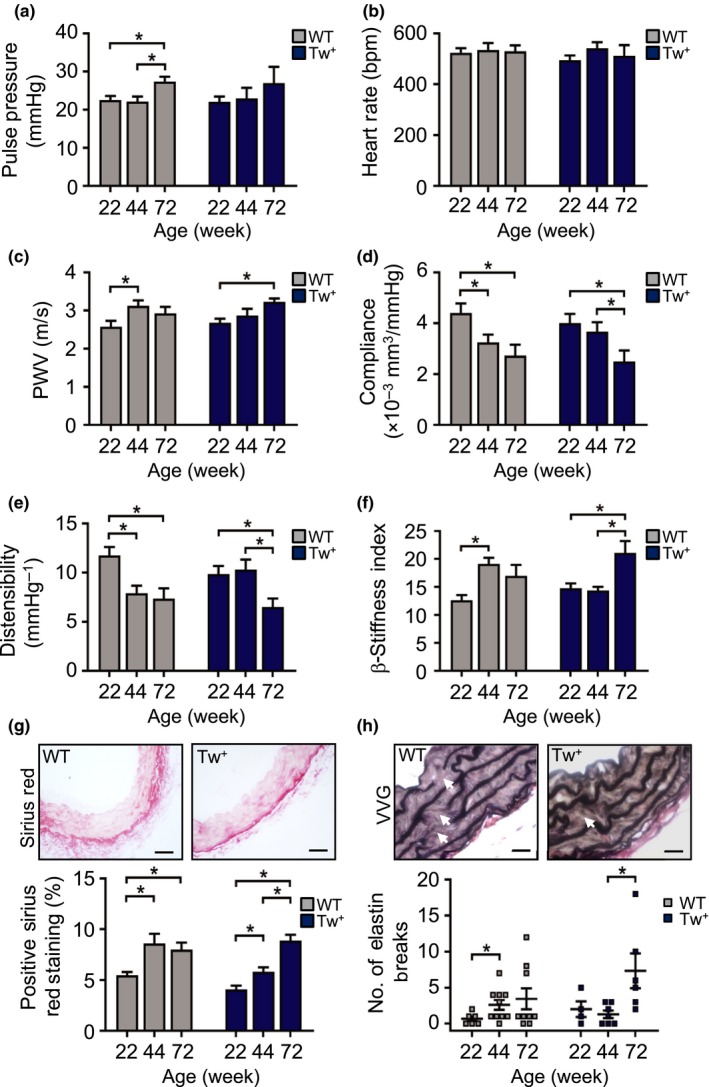
Tw^+^ mice have delayed vascular aging. (a–f) Pulse pressure, heart rate, aortic pulse wave velocity (PWV), and carotid artery compliance, carotid artery distensibility and carotid β‐stiffness index in WT and Tw^+^ mice aged 22–72 wk (*n* = 6–9), (g) representative images of aorta from 44‐wk‐old WT and Tw^+^ mice stained with Sirius Red and quantification of the percentage positive staining (scale bar = 50 μm) and (h) representative images of aorta from 44‐wk‐old WT and Tw^+^ mice stained with Verhoeff–van Gieson (VVG) showing elastin breaks (white arrows) and quantification of the number of elastin breaks. Scale bar = 20 μm (*n* = 3–7). WT (grey labels); Tw^+^ (blue labels). Data are means ± *SEM*. *p < .05 using ANOVA with Tukey post‐test

As vascular aging markers changed significantly between 22 and 72 wk in WT mice (Figures 1 and 2), we examined parameters of vascular aging in Tw^+^ vs. WT mice aged 22–72 wk. Data are presented with WT and Tw^+^ time courses adjacent to identify acceleration or delay of vascular aging (Figure 5). Pulse pressure increased in WT mice at 72 wk, but was unchanged in Tw^+^ mice, with no differences in heart rate in either group (Figure 5a,b). Carotid artery compliance and distensibility declined while PWV and stiffness increased at 44 wk in WT mice; however, these changes were all delayed until 72 wk in Tw^+^ mice (Figure 5c–f). When comparing directly to WT, β‐stiffness, distensibility and systolic and diastolic Doppler velocity were all significantly improved at 44 wk in Tw^+^ mice. Collagen content peaked by 44 wk in WT mice, whereas peak collagen content was delayed to 72 wk in Tw^+^ mice (Figure 5g). Increased elastin breaks were seen at 44 wk in WT mice, but delayed in Tw^+^ mice to 72 wk (Figure 5h). These data demonstrate that augmenting mtCN enhances mitochondrial respiration and delays both physiological and structural changes associated with vascular aging.

To better understand how Twinkle expression delays vascular aging, we examined regulators associated with mtCN, mitochondrial mass and autophagy. Tw^+^ mice showed increased TFAM expression at 44 wk compared to WT, but this difference was no longer present at 72 wk (Figure S6a). PGC1α levels were higher in Tw^+^ mice at 72 wk compared to WT, consistent with a potential increase in mitochondrial biogenesis in Tw^+^ mice (Figure S6b). Furthermore, levels of the autophagy marker p62 were reduced in Tw^+^ mice at 44 wk, but similar to WT at 72 wk (Figure S6c).

### 2.5 Effects of PolG mutation on mitochondrial respiration and vascular aging markers

**Figure 6:**
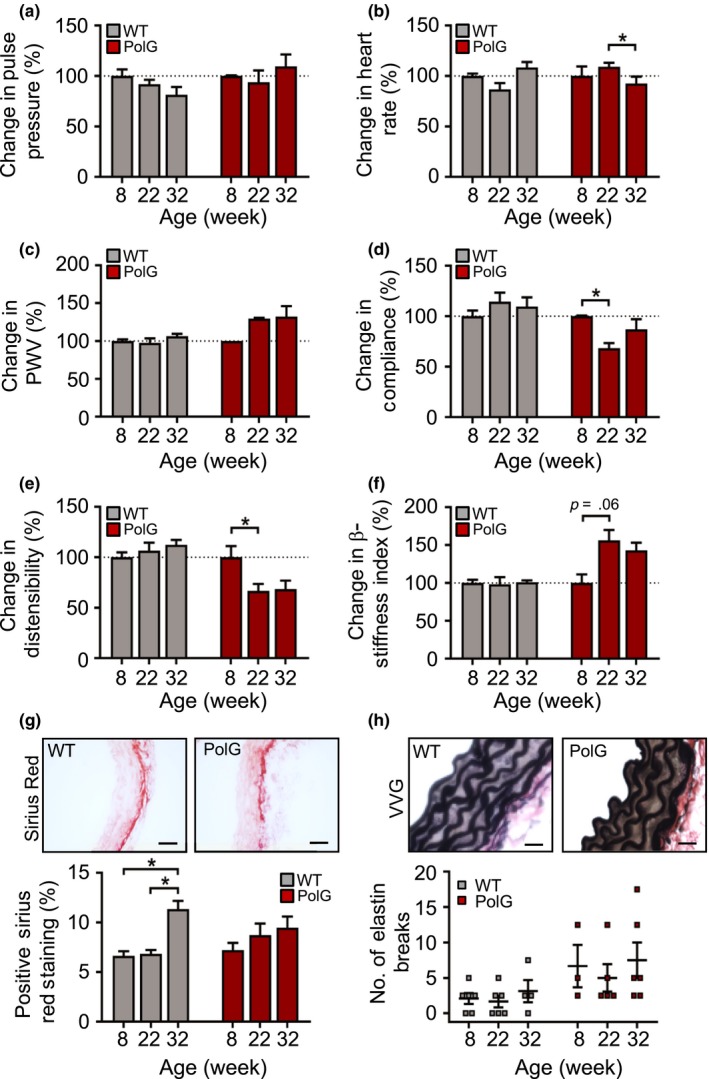
PolG mice have accelerated vascular aging. (a–f) Pulse pressure, heart rate, aortic pulse wave velocity (PWV) and carotid artery compliance, carotid distensibility and carotid β‐stiffness index in WT and PolG mice aged 8–32 wk relative to 8‐wk‐old mice (*n* = 5–9), (g) representative images of aortas from 22‐wk‐old WT and PolG mice stained with Sirius Red and quantification of positive staining (scale bar = 50 μm) and (h) representative images of aortas from 8‐wk‐old WT and PolG mice stained with Verhoeff–van Gieson (VVG) for measuring elastin breaks (scale bar = 20 μm) (*n* = 4–6). WT (grey labels); PolG (red labels). Data are means ± *SEM*. *p < .05 using ANOVA with Tukey post‐test

To confirm that changes to mtDNA can directly promote vascular aging, we examined vascular aging markers in mice with compromised mtDNA integrity. PolG^mut/mut^ mice (hereafter denoted as PolG mice) have an induced homozygous aspartic acid to alanine mutation of the exonuclease domain of the nuclear‐encoded mitochondrial PolG. The resulting defect in proofreading function results in widespread mtDNA point mutations and deletions (Trifunovic et al., 2004). Indeed, PolG mice show extensive mtDNA damage and reduced mitochondrial respiration in their aortas and cultured VSMCs, with no change in ROS early in life (Yu et al., 2013). PolG mice showed multiple features of progeria, including kyphosis and greying of fur, and became too frail to survive instrumentation beyond 32 wk; we therefore examined vascular aging markers between 8 and 32 wk of age in both WT and PolG mice. In addition, PolG mice had significantly lower body weights particularly at later time points; we therefore compared all measurements against 8‐wk‐old mice in each group to avoid systemic confounders. PolG mice showed no significant changes in pulse pressure and aortic PWV with a minor reduction in heart rate by 32 wk (Figure 6a–c). In contrast, there was a marked reduction in carotid artery compliance and distensibility by 22 wk in PolG mice, and a borderline increase in β‐stiffness index (*p* = .06), which occurred earlier than in WT mice (Figure 6d–f). Interestingly, WT mice had increased aortic collagen by 32 wk, whereas PolG mouse aortas showed no significant changes, and elastin breaks did not change significantly in either group over this shorter time course (Figure 6g–h). These data suggest that mitochondrial damage and dysfunction accelerate physiological markers of vascular aging before features of structural aging are apparent.

## 3 DISCUSSION

**Figure 7:**
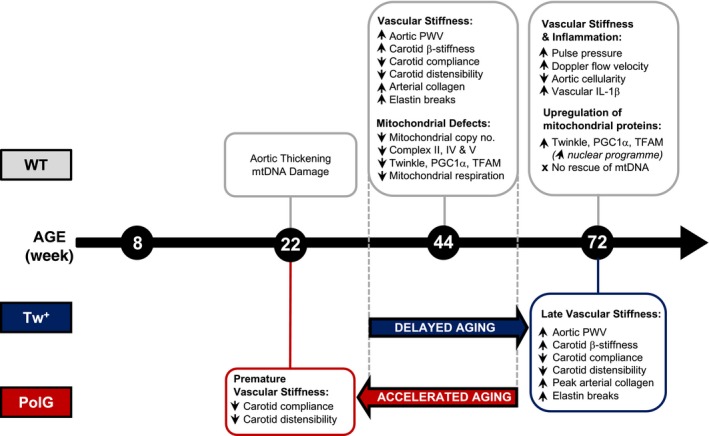
Changes in WT, Tw^+^ and PolG mice during vascular aging. Summary of the age at which changes are first observed over 8–72 wk of age in WT, Tw^+^ and PolG mice. mtDNA, mitochondrial DNA; mtCN, mitochondrial copy number; PWV, pulse wave velocity

Aging represents the largest risk factor for the development of cardiovascular disease, identifying an important need for accurate techniques and parameters in vascular aging research. We studied how and when vascular aging develops in mice, and how manipulations to mtDNA and mitochondrial function affect vascular aging. There are a number of important findings in our study. First, we show that vascular aging in mice can be demonstrated by changes in a variety of physiological parameters, with multiple robust reproducible markers appearing as early as 44 wk. These parameters recapitulate those in humans, although their sensitivity to detect aging differs. Our study provides standardized early and reproducible time points, methods and physiological parameters to study vascular aging in mice. Second, we show that mouse vascular aging is associated with characteristic structural changes over the same time, confirming that these changes in physiological parameters represent structural changes associated with aging. Third, we show that mtCN, the proteins that regulate mtCN, and mitochondrial respiration are all reduced at the same age that changes in functional and structural parameters were observed. Finally, using gain‐ and loss‐of‐mitochondrial‐function mouse models, we identify that mtCN and mtDNA integrity directly regulate the onset and progression of vascular aging in mice. The time course of changes in vascular aging markers and mitochondrial CN, DNA damage, regulatory protein expression and function are summarized in Figure 7.

Improved animal welfare means that laboratory mice can now live more than 2 years. This complicates aging research as age‐associated multi‐organ pathologies can render such aged mice too frail for functional analyses, and makes aging studies very long and expensive to perform. There is also little consensus as to when mice develop the earliest signs of aging. Using a range of five different ages and multiple physiological parameters, we identify an age window for the onset of vascular aging between 32 and 44 wk of age. These physiological changes occurred concurrent with changes in vascular structure that are characteristic of aging, including collagen accumulation, elastin breaks, reduced medial cellularity and vessel wall thickening. This age window is far earlier than previously described and allows for far shorter vascular aging protocols. Our study also addresses the causes vs. the consequences of vascular aging, for example whether vascular stiffening precedes or follows pulse pressure changes, a subject of recent debate (Humphrey et al., 2016). Changes in arterial compliance, distensibility, β‐stiffness index and PWV as well as structural changes were all present before changes in pulse pressure appeared, suggesting that increased pulse pressure is a consequence rather than a cause of vessel stiffening, at least at early time points in mice.

The role of mitochondrial dysfunction and ROS in aging remains controversial. The prevailing view has been that mitochondrial damage and dysfunction both occur in multiple tissues in aging and promote aging, possibly through ROS accumulation and oxidative damage. However, reduced mitochondrial respiration can result in normal or greater lifespan, such as mice heterozygous for the myosin light chain kinase gene (*mlck1*), the penultimate enzyme in ubiquinone biosynthesis, despite reduced ATP synthesis and increased mitochondrial ROS production (Hekimi, 2013). In addition, mt‐Deletor mice (which have mutant Twinkle) have large–scale mitochondrial deletions and reduced respiratory function in some tissues late in life, but a normal lifespan (Tyynismaa et al., 2004). Furthermore, some studies show that antioxidant therapies do not improve lifespan despite reducing ROS (Perez et al., 2009), and some *Caenorhabditis elegans* respiratory chain mutants show increased superoxide generation but have increased rather than decreased longevity (Feng, Bussiere & Hekimi, 2001; Yang & Hekimi, 2010). Similar observations from *Drosophila melanogaster, C. elegans* and yeast (reviewed in Wang & Hekimi, 2015) suggest that the pathways connecting mtDNA damage, respiratory dysfunction, ROS and aging are more complex than previously suggested.

To dissect the role of mtDNA‐mediated mitochondrial dysfunction in vascular aging, we examined mice with specific mtDNA manipulations that do not change vascular ROS at the time points studied (Yu et al., 2013, 2017). Both mtCN and mitochondrial respiration were reduced markedly by 44 wk in WT mice, concurrent with physiological and structural markers of vascular aging. Multiple markers of vascular aging were delayed in Tw^+^ mice or accelerated in PolG mice, indicating that manipulations that change mitochondrial respiration can regulate vascular aging independent of ROS, at least at early time points, and that mitochondrial respiration has direct regulatory effects on the rate of vascular aging. Our data suggest that the protective effect of Tw overexpression may be related to an enhanced mtCN regulation, mitochondrial biogenesis and improved autophagy.

The mechanisms responsible for the mtCN decline in aging are poorly understood. The minimal mtDNA replication machinery comprises PolG, mtSSB and Twinkle (Korhonen, Pham, Pellegrini & Falkenberg, 2004). Although PolG defects cause mtDNA damage, PolG alone cannot replicate mtDNA (Korhonen et al., 2004), and PolG overexpression does not increase mtCN in cell lines (Schultz et al., 1998). In contrast, *mtSSB* mRNA expression parallels mtDNA abundance in mammalian tissues and is upregulated with mitochondrial biogenesis (Schultz et al., 1998). TFAM and Twinkle are the two main regulators of mtCN, which is directly proportional to expression levels of both proteins (Ekstrand et al., 2004; Tyynismaa et al., 2004). TFAM also stabilizes the nucleoid structure of mtDNA and initiates replication (Kaufman et al., 2007). We find a marked reduction in mtCN, expression of TFAM, Twinkle and PGC1α, and mitochondrial respiratory complexes at 44 wk. Remarkably, despite this reduction at 44 wk, TFAM, Twinkle and PGC1α increased again by 72 wk, which may be a compensatory mechanism to recover mtCN levels, albeit insufficient to restore mtCN and respiratory function. Similar reductions in mtCN and compensatory responses have been shown in mouse glial neurons at 40–52 wk of age (Stauch, Purnell & Fox, 2014) or rat brain (Picca et al., 2013). Interestingly, TFAM, Twinkle and PGC1α are all nuclear‐encoded, as is complex II, which was the only complex to have significantly enhanced levels from 44 to 72 wk, suggesting that the nuclear genome may increase proteins necessary for enhancing mtCN to recover mitochondrial function, but that these are insufficient to increase mtCN or rescue the respiratory deficit.

Our study has a number of limitations. First, it is possible that PolG and Tw^+^ mice do not recapitulate the same mechanisms that accelerate or protect against loss of mitochondrial function seen in normal aging, respectively. In particular, the rate at which mtDNA mutations reach phenotypic expression differs markedly among tissues (Vermulst et al., 2008) and vessels might be protected from these manipulations. However, aging at/after 44 wk was associated with a marked reduction in mtCN and mitochondrial respiration, while Tw^+^ mice maintained mtCN and respiration at 44 and 72 wk to levels seen in younger mice. Second, the cause of reduced mtCN with age is not known, but our data suggest reductions in Twinkle and TFAM and the upstream regulator PGC1α are important contributors. However, despite the compensatory rise in PGC1α, TFAM and Twinkle at 72 wk, it appears that age‐related mtCN depletion becomes irreversible. Our data also do not prove that reduced mtCN directly reduces mitochondrial respiration. However, there was a reduction in mitochondrial OCR with no change in ECAR with increasing age associated with reduced mtCN, and Tw^+^ mice showed increased mtCN and increased respiration, suggesting a causal relationship. Third, the models used regulate mtDNA replication and replicative proofreading primarily and may have systemic effects that might affect vascular physiological parameters; however, there were no changes in heart rate or mean systolic or diastolic blood pressures in Tw^+^ or PolG mice, particularly at time points when changes in vascular physiological parameters were first apparent. Fourth, our assays were performed on whole arteries and we have not determined which cell type(s) in the vessel wall is/are primarily responsible for the multiple physiological and structural changes observed. However, VSMCs comprise the bulk of the mouse artery and synthesize collagen and elastin; it is therefore likely that VSMCs are responsible for the reduced mtCN and respiration as well as changes in passive physiological parameters and vascular structure. Furthermore, other studies suggest that mice do not develop EC dysfunction over the time course used in our study (Modrick, Kinzenbaw, Chu, Sigmund & Faraci, 2012). Finally, some of the changes observed appear small, and their biological significance as risk factors for cardiovascular disease is unproven; however, the 1.3‐fold reduction in compliance and distensibility, 1.5‐fold increase in β‐stiffness index and 1.3‐fold increase in PWV are similar to changes seen in human aging (reviewed in Vlachopoulos et al., 2010).

In summary, we have identified standardized time points and a combination of biologically coherent physiological parameters to measure vascular aging in mice, and show that the first physiological and structural changes are apparent by 44 wk. Reduced mitochondrial respiration is not only present as mouse arteries age, but manipulations that result in increased or decreased respiration delay or accelerate changes associated with aging, respectively. Our data highlight the importance of mitochondrial function in vessel aging, provide novel insight into the interaction between mtCN and mitochondrial respiration and illustrate the importance of preserving the mitochondrial genome to maintain healthy vessel aging.

## 4 EXPERIMENTAL PROCEDURES

### 4.1 Animals

This research was regulated under the Animals (Scientific Procedures) Act 1986 Amendment Regulations 2012 following ethical review by Cambridge University Animal Welfare and Ethical Review Body (AWERB). Homozygous knock‐in PolG mutation or heterozygous Twinkle overexpression mice have been described previously (Trifunovic et al., 2004; Tyynismaa et al., 2004). Male and female mice aged 8, 22, 44 or 72 wk (including 32 wk for PolG) were studied with age‐matched WT littermate controls used for each strain. Animals were provided chow diet and water ad libitum and maintained under a 12:12‐hr light–dark cycle.

### 4.2 Imaging and blood pressure measurements

Where possible, the experimenter was blinded to mouse genotype, and WT controls and transgenic mice were always studied on the same day. Mice were anaesthetized with inhaled isoflurane (2.5% in 1.5 L/min O_2_; maintained at 1.5%) and placed supine on a heated platform. Right common carotid artery (RCCA) M‐mode vascular diameters and pulsed Doppler velocities were obtained at an angle of 50–59° using a 30‐MHz probe (Vevo 770; Fujifilm VisualSonics, Amsterdam, The Netherlands). Immediately following imaging, the RCCA was cannulated with 1.2F blood pressure catheter (Transonic Scisense Inc, Ontario, Canada). Combined RCCA measurements and blood pressure generated functional indices of vascular aging including the following:

1. Compliance = Ds–Dd/Ps–Pd
2. β‐Stiffness Index = lnPs–Pd/[(Ds–Dd)/Dd]
3. Distensibility = (Ds–Dd)/(Dd*Ps–Pd)

where Ds is systolic diameter, Dd is diastolic diameter, Ps is systolic pressure, Pd is diastolic pressure and ln is the natural log (Figure S1).

Pulse wave velocity was measured by inserting a dual‐sensor catheter (1.2F; Transonic Scisense Inc, Ontario, Canada) via the left femoral artery to record blood pressure from the thoracic and abdominal aorta simultaneously (Figure S2). PWV was calculated as the distance between the pressure sensors (30 mm)/average transit time for pressure wave between sensors over 10 waves using LabScribe2 software (iWorx Systems Inc, Dover, NH). All blood pressure measurements were recorded for 10 min to reach steady state with body temperature maintained at 37.0 ± 0.2°C. Animals were sacrificed by CO_2_ inhalation and cervical dislocation, with subsequent rapid snap‐freezing of aortic tissue.

### 4.3 Histochemistry and Immunohistochemistry

Five‐micrometre transverse aorta sections were stained with Sirius Red to assess collagen expression, Verhoeff–van Gieson (VVG) for elastin integrity, or haematoxylin and eosin (H&E) for cellularity and wall thickness. Antibodies for inflammatory markers were IL‐1β (1:200; ab9722; Abcam, UK) and VCAM‐1 (1:1000; ab134047; Abcam, UK) and MAC3 (1:400; 553322; BD). In Sirius Red sections, five random evenly distributed areas were photographed at 400× magnification using a bright‐field microscope and imaging software (Image‐Pro Insight 9.1, Media Cybernetics, MD, USA). The arterial media was delineated and the percentage of media positive for red staining assessed by thresholding using ImageJ software *(*National Institutes of Health, MD, USA*)*, and averaged over the five areas. VVG‐stained sections were examined at 200× and 400× and elastin breaks defined as a disruption in the continuity of the lamina where both ends of the break were visible. H&E sections were photographed at 100× and medial cellularity determined as number of nuclei/area. Wall thickness was measured as distance between the lumen and external elastic lamina at five random points using ImageJ (NIH, MD, USA). Inflammatory markers were quantified by counting the total DAB‐positive staining relative to total cells using ImageJ (NIH, MD, USA).

### 4.4 Relative mitochondrial copy number

Abdominal aorta mtCN was estimated by qPCR on total DNA extracted using the DNeasy Blood and Tissue Kit (Qiagen Ltd, UK). Primer sequences for the mitochondrial segment were as follows: (F) GCCAGCCTGACCCATAGCCATAAT and (R) GCCGGCTGCGTATTCTACGTTA. Primer sequences for the single‐copy nuclear control were as follows: (F) TTGAGACTGTGATTGGCAATGCCT and (R) CCAGAAATGCTGGGCGCTCACT. mtCN was calculated relative to nuclear DNA using the following equations:

1. Δ*C*_T_ = mitochondrial *C*_T_ – nuclear *C*_T_
2. Relative mitochondrial DNA content = 2 × 2^−ΔCT^

### 4.5 Mitochondrial DNA damage and common deletion assay

Long and short mitochondrial segments were amplified from 15 ng total DNA using the Long Amplification Taq Polymerase kit (Takara Bio Inc, Kusatsu, Japan). Long‐mitochondrial‐segment (10.06 kb) primer sequences were as follows: (F) GCCAGCCTGACCCATAGCCATAAT and (R) GAGAGATTTTATGGGTGTAATGCGG. Short‐mitochondrial‐segment (117 bp) primer sequences were as follows: (F) GCCAGCCTGACCCATAGCCATAAT and (R) GCCGGCTGCGTATTCTACGTTA. All reactions were heat‐started at 70°C for 3 min before addition of the Taq polymerase. Long‐segment conditions were as follows: 16 cycles of (94°C for 15 s; 64°C for 12 min) with a final extension step of 72°C for 10 min. Short‐segment conditions were as follows: 23 cycles of (94°C for 30 s; 64°C for 45 s; 72°C for 45 s) with a final extension step of 72°C for 10 min. Samples were run on a 0.8% agarose gel to confirm product size only. The quantification of the samples was performed using PicoGreen labelling according to the manufacturer's protocol and using dilutions of known λ DNA standards to generate a standard curve (Thermo Fisher Scientific, UK). Fluorescence measurements were assessed using a microplate reader at an excitation of 485 nm and emission of 528 nm and were corrected for background by subtracting the fluorescence from a blank no‐template control. Long‐ and short‐segment reactions were stopped in the linear phase, and this was confirmed by running controls containing 50% of the starting undamaged template DNA which resulted in 40%–60% of the amplification. Long‐segment amplification was normalized to the short segment to account for changes in mtCN, and the estimated relative number of lesions calculated using the following formula based on the zero‐class Poisson expression as described previously (Furda, Santos, Meyer & Van Houten, 2014): Lesion Frequency =  −ln (A_D_/A_C_), where A_D_ is the amplification of the unknown sample and A_C_ is the amplification of an undamaged control. The mouse equivalent of the common 4977‐bp deletion in humans was also assessed using qPCR as described previously (Mercer et al., 2010).

### 4.6 Western blotting

Frozen mouse thoracic aortas were homogenized by manual crushing with a pestle, and then high‐speed bead shaking (TissueLyser LT; Qiagen Ltd, UK*)* in RIPA lysis buffer supplemented with protease inhibitors followed by sonication. 15 μg protein was loaded into a 4%–12% gradient gel and detected with the following antibodies: Twinkle (1:1000; Aviva Systems Biology, CA, USA), TFAM (1:500; Proteintech, IL, USA), PGC1α (1:1000; Abcam, UK), Total OXPHOS cocktail (1:500; ab110413; Abcam, UK), p62 (1:1000; Abcam, UK), Citrate Synthase (1:1000; ab96600; Abcam, UK) and β‐actin (1:5000; Sigma‐Aldrich, UK). Citrate synthase was selected as a loading control for respiratory complexes as it is mitochondrion‐specific and the expression did not change with age (further confirmed with β‐actin). Protein bands were labelled with Amersham ECL horseradish peroxidase‐conjugated secondary antibodies (GE Healthcare Life Sciences, UK) and band density quantified using ImageJ software (NIH, MD, USA).

### 4.7 Seahorse assay

Thoracic aortas were freshly harvested, dissected free of surrounding fat tissue and cut into five rings each weighing approximately 500 μg. Each ring was secured into wells of an XFe24 islet capture microplate using the mesh rings provided, washed once with bicarbonate‐free DMEM (Sigma‐Aldrich, UK) and run in a Seahorse XFe24 Extracellular Flux Bioanalyzer (Agilent Technologies, CA, USA). Mitochondrial respiratory complex inhibitors and the uncoupler FCCP were prepared for automated injection at final concentrations of 10 μg/ml oligomycin A, 1 μmol/L FCCP, 10 μmol/L antimycin A and 10 μmol/L rotenone. OCR and ECAR were measured at baseline and after the inhibitor addition, and data were normalized to individual tissue weights.

### 4.8 qPCR

RNA was extracted from whole aortas using the miRNeasy Mini Kit (Qiagen Ltd, UK) and cDNA synthesized from 1 μg of RNA using the Omniscript Reverse Transcription Kit (Qiagen Ltd, UK). 20 ng cDNA was used with 1X Rotor‐Gene SYBR Green PCR Kit (Qiagen Ltd, UK) and 0.5 μM primers in a final reaction volume of 20 μl. Cycle conditions were 95°C for 5 min, followed by 40 cycles of 95°C for 5 s and 60°C for 10 s. Primer sequences were as follows: *Twinkle* (F) GCCACGTGACTCTGGTCATTC and (R) CCATCAAAGCGATTCTTGGACA; *TFAM* (F) CAAGTCAGCTGATGGGTATGG and (R) TTTCCCTGAGCCGAATCATCC; and β*‐actin* (F) GGCACCACACCTTCTACAATG and (R) GTGGTGGTGAAGCTGTAGCC. Relative gene expression was calculated using the 2^‐ΔΔCt^ method.

### 4.9 Statistical analysis

Data were assessed for normal distribution using the D'Agostino–Pearson or Shapiro–Wilk normality test in GraphPad Prism Software 7 (GraphPad Software Inc, CA, USA). All data were analysed using either a Student's unpaired *t* test or multiple comparisons ANOVA with Tukey post‐tests. All data are expressed as mean ± *SEM*. Data represent mouse numbers or independent experiments. Statistical significance was accepted as *p* < .05.

## CONFLICT OF INTEREST

The authors declare no conflict of interest.

## AUTHOR CONTRIBUTIONS

KF and MRB designed the research. KF, JR, EPKY, NLF and AF performed the research. KF and JR analysed the data. KF, MPM and MRB wrote the manuscript.
