# Supplementary material for: Restoring mitochondrial DNA copy number preserves mitochondrial function and delays vascular aging in mice
Source: Aging Cell. 2018 May 9;17(4):e12773. doi: 10.1111/acel.12773 (PMC6052475; doi:10.1111/acel.12773)
Supplement: Supplementary file 1 [file ACEL-17-na-s001.pdf]

Supporting Information

Figure S1. Ultrasound imaging of carotid artery in mice
Cross section (left) and longitudinal (below right) ultrasound images of the mouse common carotid artery and bifurcation. RCCA; right common carotid artery, LCCA; left common carotid artery. Scale bars 1 mm. M-mode (below left) of mouse right common carotid artery demonstrating systolic and diastolic diameters.

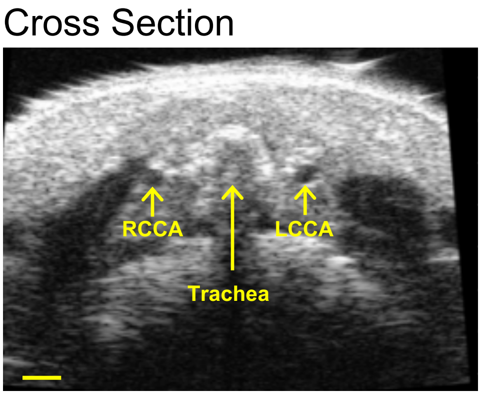

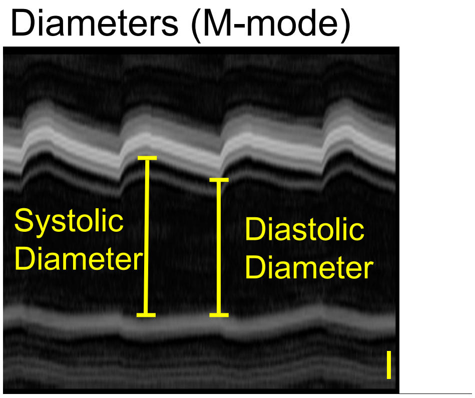

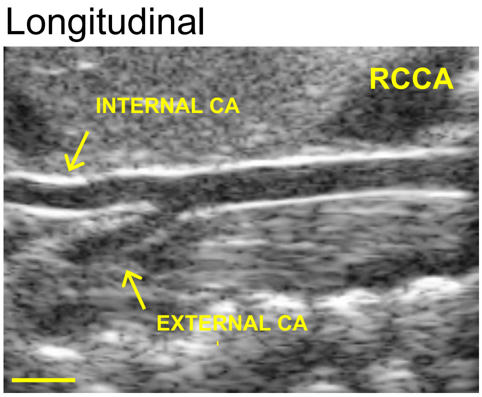

Figure S2. Measurement of pulse wave velocity in mice using a dual sensor pressure catheter
A dual sensor pressure catheter is inserted into the aorta via the left femoral artery, with simultaneous recordings taken from the descending thoracic aorta (blue circle; proximal sensor S1) and the abdominal aorta (green circle; distal sensor S2). PWV is calculated using the transit time (t) between pressure waves at the two sites and the distance (D) between sensors.

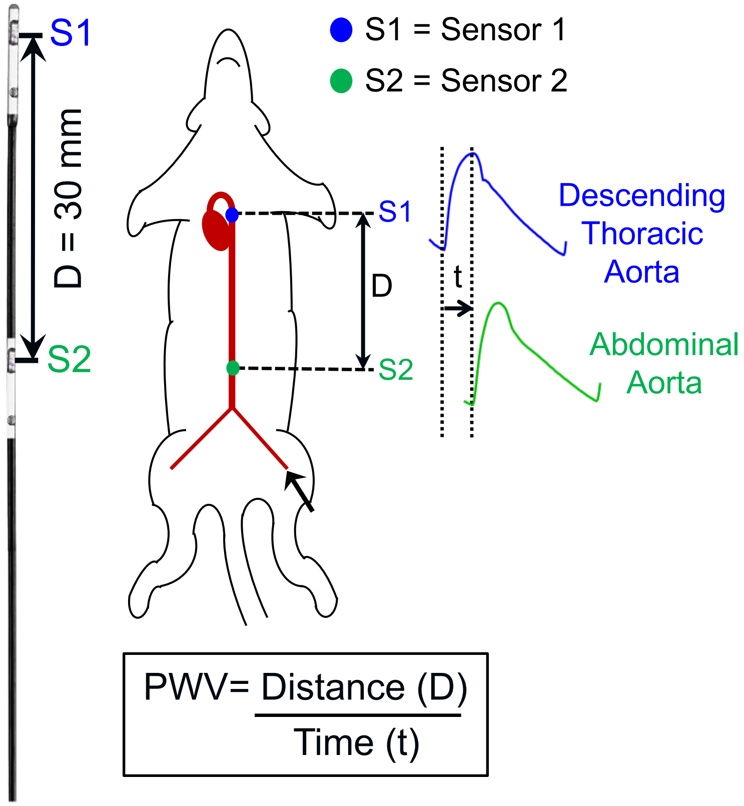

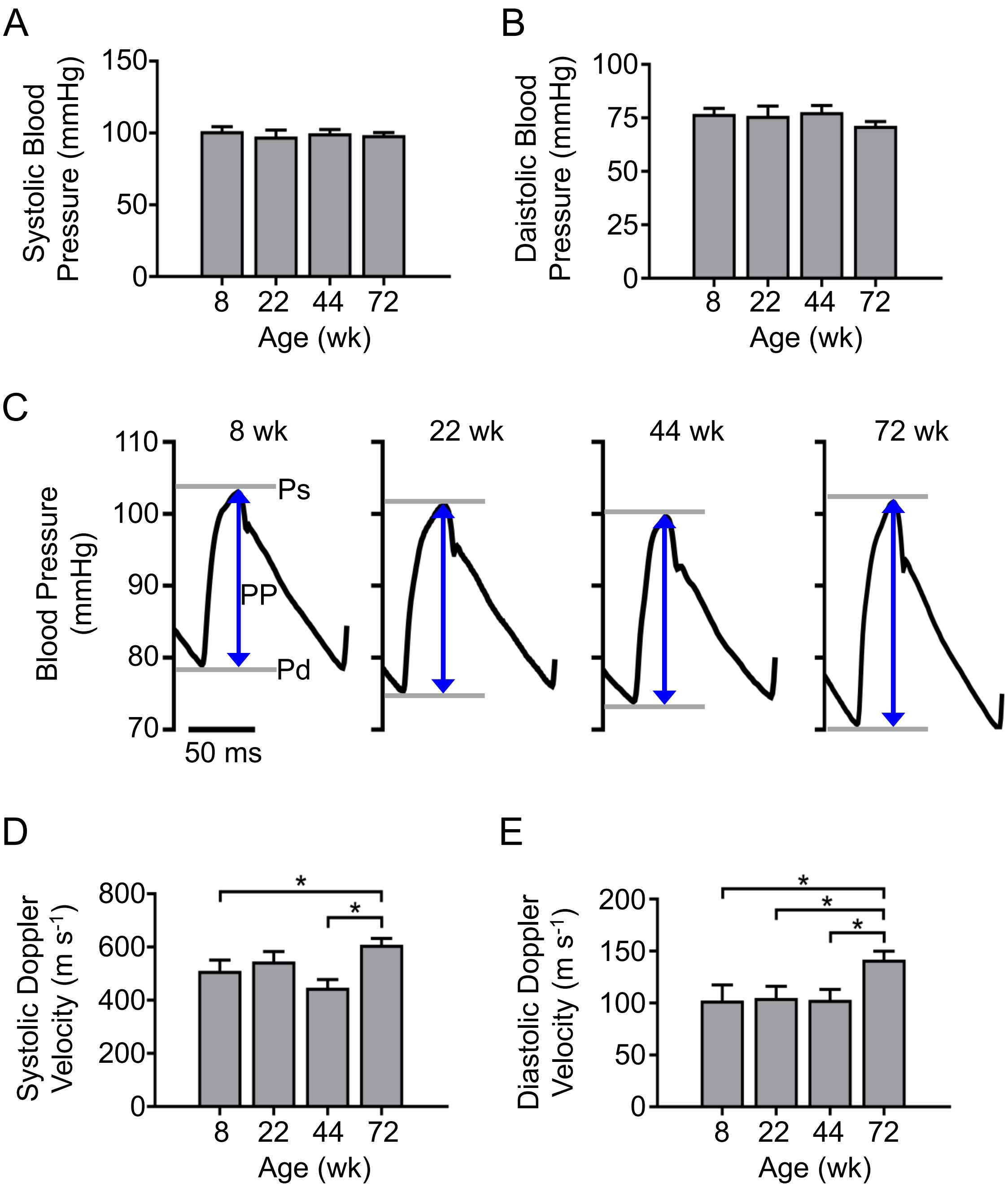

Figure S3. Blood pressure, pulse pressure and Doppler velocities in mice aged 8-72 wk
(A-B) Mean systolic and diastolic pressures, **(C)** Representative pressure traces indicating peak systolic pressure (Ps), diastolic pressure (Pd), and pulse pressure (PP = Ps-Pd; blue arrows), and **(D-E)** Doppler flow velocities in WT mice aged 8-72 wk (n=10-17). Data are means ± SEM. *$p<0.05$ using ANOVA with Tukey post-test.

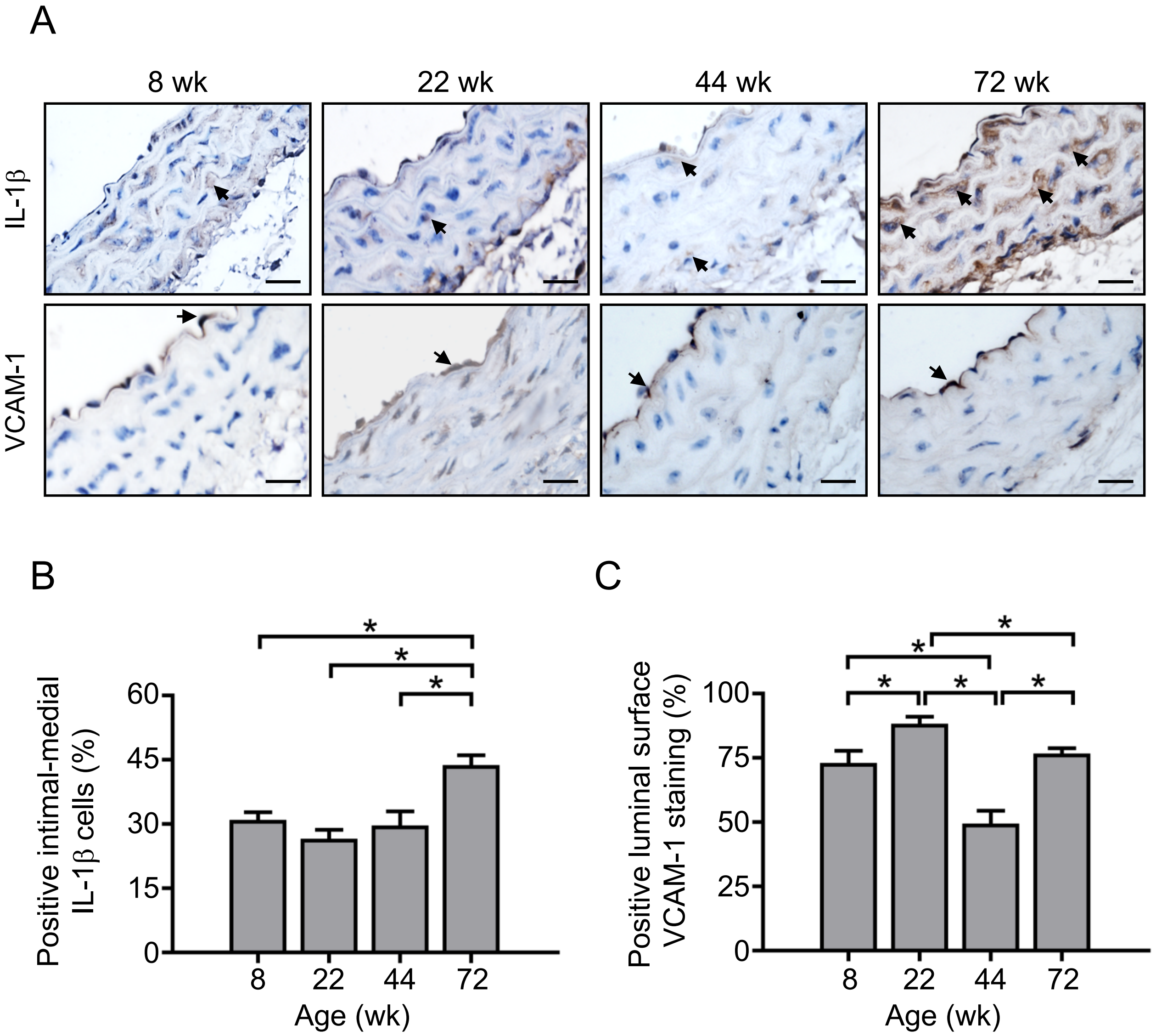

Figure S4. Markers of inflammation in aortas from mice aged 8-72 wk

(A) Representative images of aortas from WT mice aged 8-72 wk stained for IL-1β or VCAM-1, and **(B-C)** associated quantification of positive staining. Scale bars 25 μM. Black arrows indicate positive staining (n=3-4 mice). Data are means ± SEM. *p<0.05 using ANOVA with Tukey post-test.

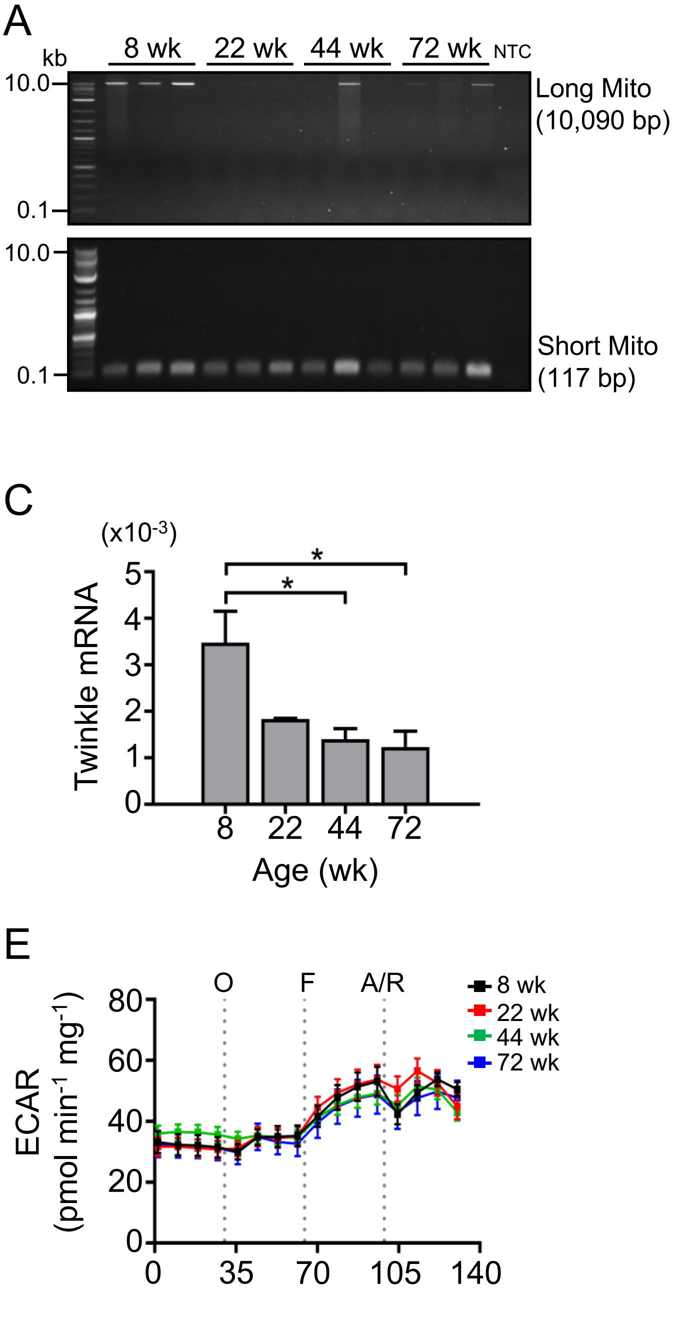

Figure S5. MtDNA damage, gene expression of mtCN regulators and ECAR in aortas from mice aged 8-72 wk

(A) Long and short segment mtDNA PCR products from 8-72 wk aortas run on 0.8% agarose gel for size confirmation only. NTC; no template control, **(B)** qPCR for the mtDNA 'common' deletion in aortas from mice aged 8-72 wk, **(C-D)** Quantification of *Twinkle* and *TFAM* mRNA in WT mice aged 8-72 wk (n=2-3 mice) and **(E-F)** Extracellular acidification rate (ECAR) indicating injection of the compounds (grey dotted lines) oligomycin (O), FCCP (F) and antimycin A and rotenone (A/R) with associated quantification after FCCP in mice from 8-72 wk (n=3 mice). Data are means ± SEM. *p<0.05 using ANOVA with Tukey post-test.

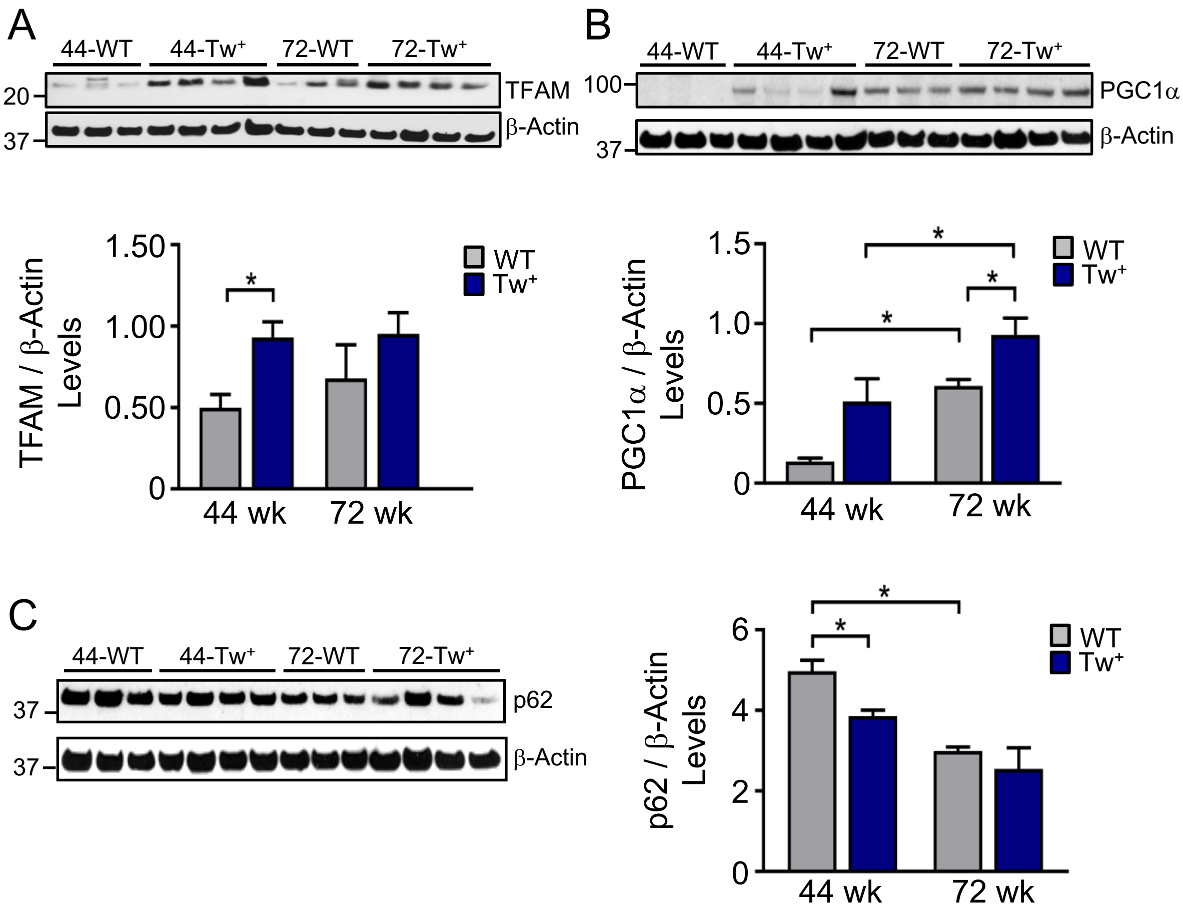

Figure S6. Tw⁺ mice have increased vascular TFAM, increased PGC1α and reduced p62 expression

(A) Western blot of TFAM, (**B)** PGC1α and **(C)** p62 in WT and Tw⁺ mice at 44-72 wk with quantification of respective relative levels (n=3-4). WT (grey labels); Tw⁺ (blue labels). Data are means ± SEM. *p<0.05 using ANOVA with Tukey post-test.
